# Quill Mites Parasitising African Shrikes: New Host Records and Distributional Insights Into Host and Habitat Specificity

**DOI:** 10.1155/japr/2067519

**Published:** 2026-06-08

**Authors:** Maciej Skoracki, Martin Hromada, Wanyoke Wamiti, Bozena Sikora, Jakub Z. Kosicki

**Affiliations:** ^1^ Department of Animal Morphology, Faculty of Biology, Adam Mickiewicz University, Poznań, Poland, amu.edu.pl; ^2^ Department of Ecology, Faculty of Humanities and Natural Sciences, University of Presov, Prešov, Slovakia; ^3^ Department of Nature Conservation, Faculty of Biological Sciences, University of Zielona Góra, Zielona Góra, Poland, uz.zgora.pl; ^4^ National Museums of Kenya, Nairobi, Kenya, museums.or.ke; ^5^ Department of Avian Biology and Ecology, Faculty of Biology, Adam Mickiewicz University, Poznań, Poland, amu.edu.pl

**Keywords:** Acari, birds, ectoparasites, host–parasite relationship, Laniidae, Syringophilidae

## Abstract

**Background:**

Quill mites of the family Syringophilidae are permanent ectoparasites inhabiting the lumen of avian feather quills and exhibiting high host and microhabitat specificity. Despite the broad geographic distribution and ecological diversity of shrikes (Laniidae), their syringophilid fauna has received insufficient study.

**Methods:**

We examined 288 museum specimens representing 13 shrike species (genera *Lanius*, *Urolestes* and *Corvinella*) from the Afrotropical region. Mites were extracted from feather quills, identified morphologically and analysed with respect to host associations, microhabitat preferences, prevalence and evolutionary patterns of host–parasite relationships.

**Results:**

Five syringophilid species belonging to three genera, *Syringophilopsis*, *Syringophiloidus* and *Aulonastus*, were recorded from eight host species. Several new host and locality records substantially expanded the known distribution of quill mites in Laniidae. All recorded species were oligoxenous and showed strict feather‐type specificity. Two phylogenetically distinct lineages of *Syringophilopsis* were found on closely related shrike hosts. No representatives of the subfamily Picobiinae were detected. Prevalence ranged from 2.5% to 25.0%.

**Conclusions:**

The distribution patterns of syringophilid mites associated with shrikes suggest a complex evolutionary history that cannot be explained by a single process and likely involves ancestral host tracking, secondary colonisation and lineage sorting, with some associations potentially reflecting cospeciation. Additionally, the absence of representatives of Picobiinae may reflect historical constraints in host–parasite associations or limited colonisation and persistence of body‐feather specialists in shrikes. All recorded species exhibited strict feather‐type specificity, highlighting microhabitat specialisation as a key factor structuring shrike–quill mite associations. The generally low prevalence observed in shrikes is likely related to their predominantly solitary behaviour, which may limit opportunities for direct transmission of these permanent and highly host‐specific parasites. These findings provide a basis for future studies on the coevolutionary relationships between corvid birds and their syringophilid mites.

## 1. Introduction

Mites of the family Syringophilidae are elongate, permanent ectoparasites of birds, measuring approximately 400–2500 *μ*m in body length. They inhabit the lumen of feather quills in the various plumage regions, including the primaries, secondaries, alulars, coverts, rectrices and body feathers [1, 2]. The family is divided into two subfamilies: Syringophilinae Lavoipierre and Picobiinae Johnston and Kethley, and members of these subfamilies differ markedly in their microhabitat preferences. Picobiines are predominantly restricted to body feathers, whereas syringophilines primarily occupy different types of flight feathers. This separation across feather types is considered to reflect an early evolutionary divergence within Syringophilidae [3–5]. A single avian host may harbour one to four species of syringophilid mites, typically representing different genera. However, each mite species is confined to a strictly defined feather type, distinct from those occupied by co‐occurring species, indicating pronounced microhabitat segregation (niche restriction) [6–10]. At present, the family comprises approximately 400 described species assigned to 62 genera, with representatives recorded from 95 avian families across 24 orders [11, 12].

The family Laniidae belongs to the corvid radiation of oscine passerines and is generally placed within the ‘crown corvids’. Molecular phylogenetic studies most frequently recover Laniidae as sister to Corvidae, consistently supporting its position within the higher corvid clade [13–15]. Ecologically and morphologically, shrikes represent a distinctive lineage of predatory passerines. Convergent with small raptors, they possess a robust, hooked bill and strong feet, enabling them to capture vertebrates and large insects [16–18]. They typically inhabit open and semiopen environments such as grasslands, savannas, semiarid regions and open woodlands, where elevated perches are available for hunting and prey caching [18, 19]. Shrikes are primarily distributed throughout Eurasia and Africa, with only two species breeding in North America. The family is absent from South America and Australia, although one species extends into New Guinea [20–22].

The first record of syringophilid mites associated with true shrikes was provided by Skoracki et al. [23], who described *Syringophiloidus weiszii* Skoracki et al., 2001, from *Lanius excubitor* Linnaeus in Slovakia. Subsequent investigations [2, 24, 25] resulted in the description of four additional species: *Syringophilopsis yosefi* Skoracki et al., 2002; *Syringophilopsis kristini* Skoracki et al., 2002; *Syringophilopsis corvinae* Skoracki and Sikora, 2003; and *Aulonastus lanius* Skoracki, 2011. These mites were recorded from three shrike hosts: *Lanius senator*, *Lanius minor* and *Corvinella corvina*, in both the Palaearctic (Europe) and Afrotropical (Cameroon) regions (see Table 1). Despite the wide geographic distribution and ecological diversity of Laniidae, their syringophilid fauna remains insufficiently explored. To date, only a small proportion of shrike species have been examined for quill mites, suggesting that the true diversity and host associations of Syringophilidae within this family are likely underestimated.

**Table 1:** Quill mites of the family Syringophilidae associated with the true shrikes (Laniidae), with their habitat and distribution.

Quill mite species	Host species	Distribution	Habitat	References
*Syringophilopsis corvinae*	*Corvinella corvina*	Afro: Uganda, Kenya and Cameroon	Ter	[25]; PP
	*Urolestes melanoleucus*	Afro: Tanzania	Ter	PP
*Syringophilopsis yosefi*	*Lanius cabanisi*	Afro: Kenya	Ter	PP
	*Lanius collaris*	Afro: Kenya	Ter	PP
	*Lanius excubitoroides*	Afro: Kenya	Ter	PP
	*Lanius* sp.	Afro: Cameroon	?	[24]
*Syringophilopsis kristini*	*Lanius minor*	Afro: Kenya and Tanzania; Pala: Romania and Slovakia	Ter	[2, 24]; PP
	*Lanius isabellinus*	Afro: Kenya	Ter	PP
*Syringophiloidus weiszii*	*Lanius excubitor*	Pala: Slovakia	GC	[23]
	*Lanius collaris*	Afro: Kenya	Ter	PP
	*Lanius excubitoroides*	Afro: Tanzania	GC and UTC	PP
	*Lanius minor*	Afro: Kenya	Ter	PP
*Aulonastus lanius*	*Lanius senator*	Pala: Europe	GC	[2]
	*Lanius somalicus*	Afro: Kenya	GC and UTC	PP

Abbreviations: Afro, Afrotropical; GC, greater covert; Pala, Palaearctic; PP, present paper; Ter, tertial; UTC, undertail covert.

The present study provides a comprehensive account of the distribution of syringophilid mites associated with African shrikes. We report numerous new records, including previously undocumented host associations and novel locality data, substantially expanding current knowledge of quill mite occurrence within this avian family in the Afrotropical region. In addition to documenting host specificity patterns, we analyse microhabitat preferences of the recorded species and explore potential coevolutionary relationships between shrikes and their quill mites. Together, these findings contribute to a more complete understanding of host–parasite associations and the evolutionary processes shaping syringophilid diversity in Laniidae.

## 2. Materials and Methods

The material examined in the present study was obtained from dry study skins housed in the National Museum of Kenya (NMK) (Nairobi, Kenya). In total, 288 specimens representing 13 shrike species (genera *Lanius*, *Urolestes* and *Corvinella*) from the Afrotropical region were examined. From each avian specimen, 1 tertiary, 1 greater covert, 2–3 undertail coverts and 5–6 body feathers (2930 feathers in total) were collected. All feathers were examined under a stereomicroscope at 10–20× magnification. When syringophilid mites were detected, the infested quills were carefully dissected using a scalpel, and individual mites were extracted. Prior to mounting, specimens were softened and cleared in Nesbitt′s solution at 60°C for 1–2 h [2]. Subsequently, mites were mounted in Hoyer′s medium [26] and examined under a ZEISS Axioscope (Carl Zeiss AG, Oberkochen, Germany) light microscope equipped with differential interference contrast (DIC) optics. Mite specimens were identified based on slide‐mounted females using the diagnostic morphological characters provided in the original species descriptions. The identification was based mainly on the structure of the peritremes, the shape and ornamentation of dorsal shields, chaetotaxy and measurements of selected idiosomal setae and was supported by comparison with the available type material of the respective species. Scientific names of birds follow Clements et al. [21]. Host phylogenies were obtained from the Birds of the World Phylogeny Explorer (Tree v1.6/Taxonomy v2025) [27], accessed in R via the package ‘clootl’ [28] and its associated AvesData repository [29]. Trees were visualised as phylograms using the R package ape [30] and exported as editable vector graphics for final figure refinement. The prevalence and its exact confidence limits (Sterne′s method; confidence level = 95*%*) were computed using Quantitative Parasitology on the Web [31, 32].

Specimen depositories (mites or hosts) are cited using the following abbreviations: AMU—Adam Mickiewicz University, Department of Animal Morphology, Poznań, Poland; NMK, Nairobi, Kenya; LMEE—Laboratory and Museum of Evolutionary Ecology, Department of Ecology, University of Prešov, Prešov, Slovakia; RMCA—Royal Museum for Central Africa, Tervuren, Belgium; SMB—Šarišské Museum of Natural History, Bardejov, Slovakia; USNM—US National Insect and Mite Collection, Beltsville, Maryland, United States; and ZISP—Zoological Institute, Russian Academy of Sciences, St. Petersburg, Russia.

## 3. Results

### 3.1. Mite Species Collected From African Shrikes

In total, we examined 13 shrike species (e.g. all three genera of the family Laniidae: monotypic *Corvinella* and *Urolestes*, and 11 species of the genus *Lanius*). Syringophilid mites were recorded from eight host species and comprised five species representing three genera: *Syringophilopsis*, *Syringophiloidus* and *Aulonastus* (Table 1). No consistent diagnostic morphological differences were observed among specimens assigned to the same mite species but collected from different hosts; therefore, we found no morphological evidence supporting their separation as distinct morphospecies. Below, we provide an annotated list of the recorded mite species, including host associations, habitat and locality data.

#### 3.1.1. *Syringophilopsis corvinae* Skoracki and Sikora, 2003

This species was originally described from the yellow‐billed shrike *C. corvina* (Shaw) in Cameroon [25]. Since its original description, no additional records have been published. Herein, we provide new locality data (Uganda, Kenya) for mites collected from the type host species and a new host record for the magpie shrike *Urolestes melanoleucus* (Jardine) from Tanzania.

Type material examined. Ex *C. corvina*; Cameroon: Galim, 14 August 1968, coll. F. Puylaert (habitat unknown)—6 females (reg. no. RMCA 141.266) deposited in the RMCA.

New material examined. Ex type host species; Uganda: Kyegegwa District, Kakamari, 28 April 1914, coll. unknown (habitat: tertiary)—3 females and 1 male (reg. no. MS 26‐0221‐001) deposited in the AMU (1 female), NMK (1 female and 1 male) and LMEE (1 female). Ex same host species; Kenya: West Pokot County, Kongelai (Mount Elgon area), 28 March 1964, coll. unknown (habitat: tertiary)—10 females (reg. no. MS 26‐0221‐002) deposited in the AMU (3 females), NMK (5 females) and LMEE (2 females).

Ex *U. melanoleucus*; Tanzania: 24 October 1961, coll. John Sutton (habitat: tertiary)—3 females and 2 males (reg. no. MS 26‐0221‐003) deposited in the AMU (1 female and 1 male), NMK (1 female) and LMEE (1 female and 1 male).

#### 3.1.2. *Syringophilopsis yosefi* Skoracki, Tryjanowski and Hromada, 2002

This species was originally described from an unidentified *Lanius* specie*s* in Cameroon [24]. No additional records have been reported since its description. Herein, we provide three new host records: the long‐tailed fiscal *Lanius cabanisi* Hartert from Kenya, the southern fiscal *Lanius collaris* Linnaeus from Uganda and the grey‐backed fiscal *Lanius excubitoroides* Prévost and des Murs from Kenya.

Type material examined. Ex *Lanius* sp. (Passeriformes: Laniidae); Cameroon: Yagoua, 3 August 1971, coll. F. Puylaert (habitat unknown)—female holotype and 11 female paratypes (reg. no. RMCA 141.265), deposited in the RMCA.

New material examined. Ex *L. cabanisi*; Kenya: Taita‐Taveta County, Voi, 25 January 1931, coll. unknown (habitat: tertiary)—8 females (reg. no. MS 26‐0221‐004) deposited in the AMU (2 females), NMK (4 females) and LMEE (2 females). Ex same host species and habitat; Kenya: Makueni County, Chyulu Hills (Chyulu Camp), 7 June 1938, coll. unknown—6 females (reg. no. MS 26‐0221‐005) deposited in the AMU (1 female), NMK (3 females) and LMEE (2 females). Ex same host species; Kenya: Machakos County, Machakos, 26 February 1941, coll. unknown (habitat: tertiary)—6 females (reg. no. MS 26‐0221‐006) deposited in the AMU (1 female), NMK (3 females) and LMEE (2 females). Ex same host species and habitat; Kenya: Makueni County, Makindu, 29 September 1965, coll. unknown—12 females and 5 males (reg. no. MS 26‐0221‐007) deposited in the AMU (4 females and 2 males), NMK (4 females and 1 male) and LMEE (4 females and 2 males). Ex same host species and habitat; Kenya: Kajiado County, Loitokitok (Mount Kilimanjaro area), 19 September 1974, coll. J. Sutton—7 females and 2 males (reg. no. MS 26‐0221‐008) deposited in the AMU (2 females and 1 male), NMK (3 females) and LMEE (2 females and 1 male).

Ex *L. collaris*; Uganda: Luweero District, Bombo, 20 February 1922, coll. unknown (habitat: tertiary)—5 females (reg. no. MS 26‐0221‐009) deposited in the AMU (2 females), NMK (1 female) and LMEE (2 females).

Ex *L. excubitoroides*; Kenya: Homa Bay County, Rusinga Island (Lake Victoria), 3 April 1941, coll. unknown (habitat: tertiary)—3 females and 4 males (reg. no. MS 26‐0221‐010) deposited in the AMU (1 female and 1 male), NMK (1 female and 1 male) and LMEE (1 female and 2 males).

#### 3.1.3. *Syringophilopsis kristini* Skoracki, Tryjanowski and Hromada, 2002

This species was described from the lesser grey shrike *L. minor* Gmelin in Slovakia [24] and subsequently confirmed from the same host species in Romania [2]. Herein, we provide new locality data from eastern Africa (Kenya and Tanzania) and a new host record: the isabelline shrike, *Lanius isabellinus* Hemprich and Ehrenberg, from Kenya.

Type material examined. Ex *L. minor*; Slovakia: Kurov, 6 June 1960, coll. T. Weisz (habitat: tertiary)—female holotype, 6 female and 3 male paratypes (reg. no. MS 01‐0202‐001), deposited in the AMU, SMB and ZISP.

Nontype material examined. Ex type host species; Romania: 1901, no other data—5 females (reg. no. MS 05‐1005‐001), deposited in the AMU.

New material examined. Ex type host species; Tanzania: Kilimanjaro Region, Moshi, 8 March 1953, coll. D.B.A. Bell (habitat: tertiary)—4 females (reg. no. MS 26‐0221‐011) deposited in the AMU (2 females), NMK (1 female) and LMEE (1 female). Ex same host species and habitat; Kenya: Nairobi County, Nairobi, 16 April 1942, coll. J. Summers—4 females (reg. no. MS 26‐0221‐012) deposited in the AMU (2 females) and LMEE (2 females).

Ex *L. isabellinus*; Kenya: Taita‐Taveta County, Kasigau (Kasigau Hill area), November 1938, coll. unknown (habitat: tertiary)—9 females (reg. no. MS 26‐0221‐013) deposited in the AMU (2 females), NMK (2 females) and LMEE (5 females).

#### 3.1.4. *Syringophiloidus weiszii* Skoracki, Hromada and Tryjanowski, 2001

This species was originally described from the great grey shrike *L. excubitor* Linnaeus in Slovakia [23], and no additional records have been published since its original description. Herein, we provide three new host records: the southern fiscal *L. collaris* Linnaeus from Kenya, the grey‐backed fiscal *L. excubitoroides* Prévost and des Murs from Tanzania and the lesser grey shrike *L. minor* Gmelin from Kenya.

Type material examined. Ex *L. excubitor*; Slovakia: Bardejov, 22 March 1966, coll. T. Weisz (habitat: greater coverts)—female holotype, 21 female and 10 male paratypes (reg. no. MS 01‐0110‐001), deposited in the AMU, SMB and USNM.

New material examined. Ex *L. collaris*; Kenya: Homa Bay County, Rusinga Island (Lake Victoria), 23 February 1936, coll. unknown (habitat: greater covert)—6 females and 3 males (reg. no. MS 26‐0221‐014) deposited in the AMU (2 females and 1 male), NMK (2 females and 1 male) and LMEE (2 females and 1 male).

Ex *L. excubitoroides*; Tanzania: Iringa Region, Ruaha National Park, 31 October 1957, coll. I. H. Dillingham (habitat: greater covert and undertail covert)—9 females (reg. no. MS 26‐0221‐015) deposited in the AMU (2 females), NMK (2 females) and LMEE (5 females).

Ex *L. minor*; Kenya: West Pokot County, Kapenguria, 12 April 1933, coll. unknown (habitat: greater covert)—4 females (reg. no. MS 26‐0221‐016) deposited in the AMU (1 female), NMK (2 females) and LMEE (1 female).

#### 3.1.5. *Aulonastus lanius* Skoracki, 2011

This species was described from the Woodchat Shrike *L. senator* Linnaeus in Europe [2], and no additional records have been published since its original description. Herein, we provide a new host record for the Somali fiscal, *Lanius somalicus* Hartlaub, and a new locality—Kenya.

Type material examined. Ex *L. senator*; Europe: no other data (habitat: greater covert)—female holotype and 8 female paratypes (reg. no. MS 10‐0505‐001), deposited in the AMU and ZISP.

New material examined. Ex *L. somalicus*; Kenya: Turkana County, Lodwar, 18 April 1933, coll. unknown (habitat: greater covert, undertail covert and body feather)—4 females (reg. no. MS 26‐0221‐017) deposited in the AMU (1 female), NMK (2 females) and LMEE (1 female).

### 3.2. Prevalence

In total, 2930 feathers, including tertiaries, wing coverts, undertail coverts and body feathers, were examined from 288 bird specimens representing 13 shrike species. Syringophilid mites were detected in 20 specimens belonging to eight host species. Prevalence among infected host species ranged from 2.5% to 25%. Detailed prevalence values for each infested host species are presented in Table 2.

**Table 2:** Examined African shrikes with prevalence, confidence interval and quill mite species.

Host species (sample size)	Total no. of hosts infested (prevalence [%]; 95% confidence interval [Sterne method])	Quill mite species
*Corvinella corvina* (*N* = 22)	2 (9.1%; CI: 1.6%–27.8%)	[1]
*Urolestes melanoleucus* (*N* = 6)	1 (16.7%; CI: 0.9%–63.5%)	[1]
*Lanius cabanisi* (*N* = 28)	5 (17.9%; CI: 7.9%–35.6%)	[1]
*Lanius collaris* (*N* = 81)	2 (2.5%; CI: 0.4%–9.4%)	[1]; [4]
*Lanius collurio* (*N* = 24)	0	—
*Lanius dorsalis* Cabanis (*N* = 4)	0	—
*Lanius excubitoroides* (*N* = 46)	5 (10.9%; CI: 4.8%–22.6%)	[1]; [4]
*Lanius isabellinus* (*N* = 33)	1 (3.0%; CI: 0.2%–15.8%)	[2]
*Lanius mackinnoni* (*N* = 10)	0	—
*Lanius minor* (*N* = 12)	3 (25.0%; CI: 9.6%–49.4%)	[2]; [4]
*Lanius nubicus* (*N* = 2)	0	—
*Lanius somalicus* (*N* = 19)	1 (5.3%; CI: 0.3%–24.6%)	[3]
*Lanius souzae* (*N* = 1)	0	—

*Note:* Quill mite species: [1] *Syringophilopsis yosefi*, [2] *Syringophilopsis kristini*, [3] *Aulonastus lanius* and [4] *Syringophiloidus weiszii*.

## 4. Discussion

### 4.1. Species Associated With Shrikes

**Figure 1:**
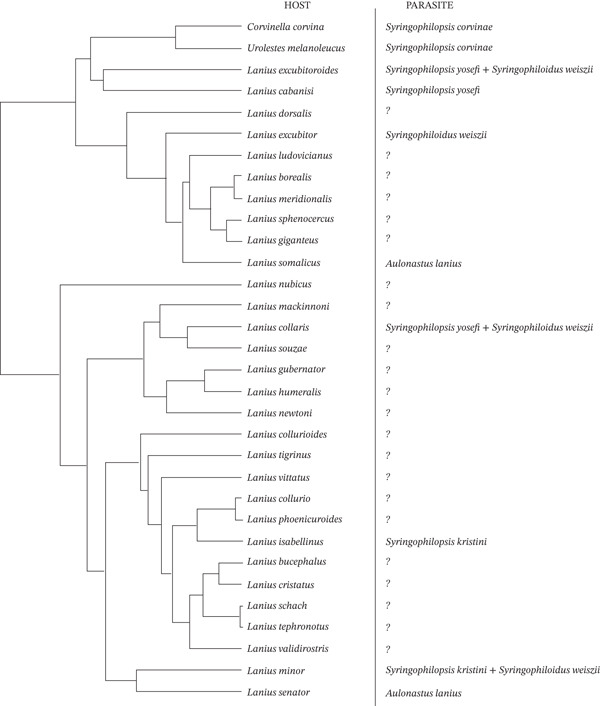
Phylogeny of the family Laniidae with the records of quill mites.

**Figure 2:**
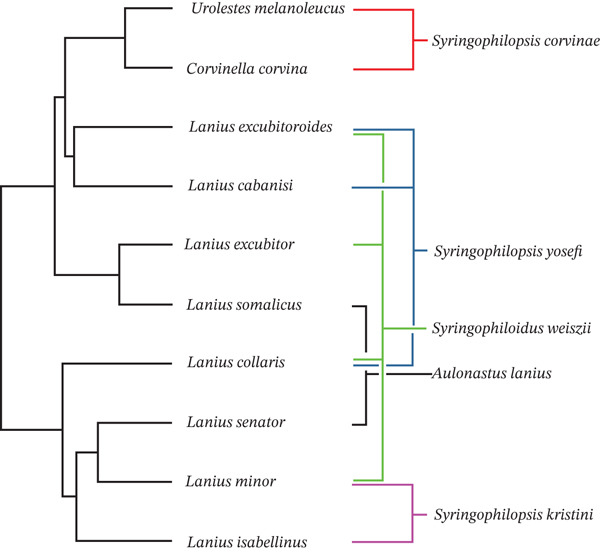
Phylogeny of the African shrikes with the records of quill mites.

The fauna of syringophilid mites associated with birds of the family Laniidae comprises five species assigned to three genera: *Aulonastus*, *Syringophiloidus* and *Syringophilopsis*. These genera are widely distributed across passeriform families [2], and their occurrence in shrikes is, therefore, not unexpected. All mite species recorded in the present study are oligoxenous, each associated with a limited number of closely related host species and restricted to the same avian genus (Table 1 and Figures 1 and 2).

*Syringophilopsis corvinae* belongs to the ‘*turdi*’ species group [2] and parasitises hosts from two closely related, monotypic genera, *Corvinella* and *Urolestes*, both endemic to Africa. This distribution is consistent with the hypothesis that the parasite colonised the common ancestor of these host lineages prior to their divergence and was subsequently retained in both descendant genera.

The second mite species belonging to the same ‘*turdi*’ clade, *S. yosefi*, is likewise restricted to African hosts. It has been recorded from two closely related species, *L. cabanisi* and *L. excubitoroides*, as well as from *L. collaris*, which is more distantly related to the former two shrike species. One plausible explanation for this distribution pattern is that several intermediate host species remain unexamined, and broader sampling may reveal a more continuous host–parasite association consistent with shared evolutionary history. Nevertheless, host switching to more distantly related shrike species cannot be excluded as an alternative explanation. A third possibility is that populations currently assigned to *S. yosefi* may represent cryptic species that are not distinguishable based on the available morphological characters. However, no consistent diagnostic morphological differences were observed among specimens from different host species; therefore, we provisionally retain them within *S. yosefi*. Future molecular data and broader host sampling will be necessary to test whether this pattern reflects a genuinely broad host range, secondary host‐switching events or cryptic diversity.

The third species of the genus *Syringophilopsis*, *S. kristini*, belongs to the ‘*elongatus*’ species group [2] and presents a more complex distribution pattern. This mite has been recorded from two shrike species, *L. minor* and *L. isabellinus*. Both hosts exhibit distributions spanning African and non‐African regions [33, 34] and belong to the same phylogenetic clade within *Lanius* [27]. The presence of two phylogenetically distinct lineages of *Syringophilopsis* (i.e. the ‘*elongatus*’ group [*S. kristini*] and the ‘*turdi*’ group [*S. corvinae* and *S. yosefi*], parasitising related host species within Laniidae) further supports the view that the syringophilid fauna of shrikes did not arise through a single cospeciation event. Under a strict cospeciation model, closely related host species would be expected to harbour closely related parasite lineages [35–38]. Instead, the occurrence of representatives of two distinct species groups on congeners of Laniidae indicates that at least two independent colonisation events may have contributed to the current host–parasite associations. One possible interpretation is that the ‘*turdi*’ lineage represents an ancestral association (present in lineages *Corvinella* and *Urolestes*) retained during the early diversification of shrikes, whereas the ‘*elongatus*’ lineage may reflect a secondary colonisation event, potentially involving host switching from another passerine lineage. Notably, colonisation by representatives of both *Syringophilopsis* species groups may have occurred even earlier in corvid evolution. Within the closely related family Corvidae, both lineages are likewise represented: *Syringophilopsis garrulus* Skoracki and Dabert, 2002 (*turdi* species group) parasitises *Garrulus glandarius* (Linnaeus), whereas *Syringophilopsis nucifragus* Skoracki, 2011 (*elongatus* species group) occurs on *Nucifraga caryocatactes* (Linnaeus). This broader distribution across corvid hosts suggests that diversification of the two *Syringophilopsis* lineages may predate the radiation of shrikes, with subsequent sorting and colonisation shaping their present associations within Laniidae.

The fourth species, *S. weiszii*, occurs on African shrikes such as *L. excubitoroides* and *L. collaris*, as well as on species whose distributions extend beyond the African continent. These include *L. excubitor*, which ranges across Europe, western Asia and northern Africa, and *L. minor*, distributed from the Iberian Peninsula through Siberia and Central Asia to sub‐Saharan Africa [33, 39–43]. The occurrence of *S. weiszii* on host species that are not all closely related may indicate a more complex evolutionary history than currently documented. One possible explanation is that many shrike species remain insufficiently examined for this parasite and that *S. weiszii* may in fact exhibit a wider host distribution than currently known. It is conceivable that this mite was associated with ancestral lineages within *Lanius* and has subsequently been retained in multiple descendant host lineages. Alternatively, the current pattern may reflect the presence of cryptic diversity within populations currently assigned to *S. weiszii*. At present, however, no consistent diagnostic morphological differences have been detected among specimens from different host species. Therefore, we provisionally interpret these records as representing a single morphologically recognisable species while emphasising that molecular data and broader host sampling are needed to test this hypothesis.

Regarding the final species, *A. lanius*, our data are more limited. In the examined material, this species was confirmed in only a single individual of *L. somalicus* (*N* = 19 examined hosts). Although the available data suggest that *A. lanius* may occur at low prevalence in shrikes, broader sampling is required before firm conclusions can be drawn about its rarity or distribution within the genus.

### 4.2. Absence of Picobiinae Mites in Shrikes

Another noteworthy finding is the complete absence of syringophilid mites of the subfamily Picobiinae in the examined material. Despite extensive screening of body feathers that typically harbour picobiines, no representatives of this subfamily were detected. This absence is particularly striking given that picobiines are widely distributed across numerous passerine families [44]. Several explanations may account for this pattern. Shrikes may never have acquired picobiine mites during their evolutionary history, either because their ancestral lineages were not parasitised by picobiines or because ecological or behavioural traits limited colonisation opportunities. Alternatively, picobiines may have been present in ancestral Laniidae but subsequently lost through lineage sorting or extinction events. Given the high degree of host specificity and microhabitat restriction characteristic of syringophilids, even subtle differences in feather structure, grooming behaviour or ecological niche could influence long‐term parasite persistence. Importantly, picobiine mites are exceedingly rare across the entire corvid clade. This specious superfamily includes 10 families [45, 46], and to date, only a single species has been recorded from representatives of the genera *Cissa* and *Urocissa* (Corvidae) and two species from birds‐of‐paradise (Paradisaeidae), one of which clearly represents a secondary host‐switching event [44, 47]. Despite extensive sampling, no picobiine mites have been documented from other corvid families, including Vireonidae, Rhipiduridae, Dicruridae, Monarchidae, Ifritidae, Corcoracidae, Melampittidae or Platylophidae. Thus, the absence of Picobiinae in Laniidae may not represent an isolated anomaly but rather a broader evolutionary pattern within Corvoidea. Although sampling limitations cannot be entirely ruled out, the consistent absence of picobiines across multiple shrike species suggests that this pattern is unlikely to be accidental. If confirmed by further studies, the shrike‐associated syringophilid fauna appears to have been assembled exclusively from lineages occupying flight‐feather niches, potentially reflecting deep historical constraints within the corvid clade.

### 4.3. Habitat Preference

All syringophilid species recorded from shrikes occupied microhabitats consistent with those previously documented for their respective genera. *Syringophilopsis corvinae*, *S. yosefi* and *S. kristini* were found exclusively in the quills of tertiary feathers, whereas *S. weiszii* and *A. lanius* inhabited tertiaries, greater coverts and undertail coverts. The observed microhabitat distribution, therefore, reflects strong feather‐type specificity at the genus level. Three shrike species, *L. minor*, *L. collaris* and *L. excubitoroides*, harboured two different syringophilid species (Table 1). In each case, the mites occupied distinct feather types, with no niche overlap. Despite the potential for co‐occurrence within a single host, no cases of simultaneous coinfestation were observed. This strict segregation likely reduces interspecific competition and may facilitate stable coexistence at the host‐species level [48]. Feather‐type specialisation appears to function as a primary ecological axis structuring syringophilid assemblages. Because different feather categories represent structurally and functionally distinct microhabitats, occupation of separate feather niches may promote ecological isolation even within a single host [2, 4]. From an evolutionary perspective, feather types can be regarded as discrete adaptive zones within the host plumage. Diversification in Syringophilidae may, therefore, be driven not only by host speciation but also by ecological differentiation within the integumentary system. The shrike‐associated fauna, characterised by strict microhabitat partitioning and absence of niche overlap, is consistent with this broader evolutionary framework.

### 4.4. Prevalence

A total of 293 shrike specimens were examined, with the largest sample obtained from *L. collaris* (*N* = 81). In this species, only 2.5% of individuals were infested with syringophilid mites. In the remaining host species, prevalence ranged from 3% to 25% (Table 2). The highest values were observed in species represented by small sample sizes and should, therefore, be interpreted with caution, as they may not accurately reflect population‐level infestation rates. For instance, in *L. excubitor*, a prevalence of 3.54% (18 infested individuals out of 508 examined) was recorded [23]. Although relatively low, this estimate is based on a comparatively large sample size and may provide a more reliable approximation of natural infestation levels. The generally low prevalence observed across shrikes may be linked to their predominantly solitary and territorial behaviour, which likely restricts opportunities for horizontal transmission of these highly host‐specific parasites. Because syringophilid mites are permanent parasites, transmission is expected to occur primarily through direct physical contact between hosts, particularly during mating or parental care. Such behavioural and life‐history constraints may, therefore, contribute substantially to the relatively low infestation rates documented in this study.

## 5. Conclusions

The present study substantially expands current knowledge of syringophilid mites associated with shrikes (Laniidae) by providing new host and locality records and documenting patterns of host specificity, microhabitat use and prevalence. The shrike‐associated fauna comprises a limited number of oligoxenous species representing three genera, yet their distribution across hosts and regions reveals a complex evolutionary history. The coexistence of phylogenetically distinct lineages of *Syringophilopsis* on closely related shrike species indicates that the parasite fauna of Laniidae did not arise through a single cospeciation event. Instead, the observed patterns are more consistent with a mosaic assembly involving ancestral host tracking, secondary colonisation events and lineage sorting. The distribution of certain mite species across both Afrotropical and Palaearctic hosts further suggests that some lineages may have parasitised early ancestors of *Lanius* prior to its diversification. The consistent absence of Picobiinae mites, despite extensive sampling, suggests that the syringophilid fauna of shrikes has been assembled exclusively from lineages occupying flight‐feather niches. Together with the strict microhabitat partitioning observed among co‐occurring species, this pattern highlights the importance of feather‐type specialisation as a key ecological and evolutionary driver in Syringophilidae. Overall, the shrike–quill mite system represents a promising model for future cophylogenetic and comparative studies aimed at disentangling the relative roles of host history, ecological constraints and parasite dispersal in shaping host–parasite associations.

## Funding

This research was financially supported by the Slovak Research and Development Agency, APVV‐22‐0440; the Scientific Grant Agency of the Ministry of Education, Science, Research and Sport of the Slovak Republic, VEGA 1/0783/26; and the Recovery and Resilience Plan of the Slovak Republic, 09I03‐03‐V06‐00052.

## Conflicts of Interest

The authors declare no conflicts of interest.

## Data Availability

The data that support the findings of this study are available from the corresponding authors upon reasonable request.

## References

[1] Kethley J. B. , A Revision of the Family Syringophilidae (Prostigmata: Acarina), Contributions of the American Entomological Institute. (1970) 6, 1–76.

[2] Skoracki M. , Quill Mites (Acari: Syringophilidae) of the Palaearctic Region, Zootaxa. (2011) 2840, no. 1, 1–414, 10.11646/zootaxa.2840.1.1.

[3] Skoracki M. , Glowska E. , and Bochkov A. V. , Phylogeny of Quill Mites of the Family Syringophilidae (Acari: Prostigmata) Based on their External Morphology, European Journal of Entomology. (2013) 110, no. 4, 663–675, 10.14411/eje.2013.090.

[4] Kethley J. B. and Johnston D. E. , Resource Tracking Patterns in Bird and Mammal Ectoparasites, Miscellaneous Publications of the Entomological Society of America. (1975) 9, 227–236, 10.4182/AHJN5941.9-5.231.

[5] Johnston D. E. and Kethley J. B. , A Numerical Phenetic Study of the Quill Mites of the Family Syringophilidae (Acari), Journal of Parasitology. (1973) 59, no. 3, 520–530, 10.2307/3278787.

[6] Kaszewska-Gilas K. , Kosicki J. Z. , Hromada M. , and Skoracki M. , Global Studies of the Host-Parasite Relationships Between Ectoparasitic Mites of the Family Syringophilidae and Birds of the Order Columbiformes, Animals. (2021) 11, no. 12, 10.3390/ani11123392, 34944169. PMC8697884 34944169

[7] Schmäschke R. , Sachse M. , Eulenberger K. , and Schöne R. , Quill Mites - Little Known Parasites of Birds, Internationalen Symposiums über die Erkrankungen der Zoo- und Wildtiere. (2003) 41, 127–133, Verhandlungsbericht des.

[8] Skoracki M. , Michalik J. , and Sikora B. , Prevalence and Habitat Preference of Quill Mites (Acari, Syringophilidae) Parasitizing Forest Passerine Birds in Poland, Acta Parasitologica. (2010) 55, no. 2, 188–193, 10.2478/s11686-010-0021-7.

[9] Skoracki M. and Hebda G. , Quill Mites (Acari: Syringophilidae) from Aegithalos Caudatus (Passeriformes: Aegithalidae), Zootaxa. (2004) 691, no. 1, 1–6, 10.11646/zootaxa.691.1.1.

[10] Marciniak-Musiał N. , Skoracki M. , Kosicki J. Z. , Unsöld M. , and Sikora B. , Host-Parasite Relationships of Quill Mites (Syringophilidae) and Parrots (Psittaciformes), Diversity. (2023) 15, no. 1, 10.3390/d15010001.

[11] Zmudzinski M. , Skoracki M. , and Sikora B. , An Updated Checklist of Quill Mites of the Family Syringophilidae (Acariformes: Prostigmata), 2023, figshare, https://figshare.com/articles/dataset/An_updated_checklist_of_quill_mites_of_the_family_Syringophilidae_Acariformes_Prostigmata_/16529574, 10.6084/m9.figshare.16529574.

[12] Glowska E. , Chrzanowski M. , and Kaszewska K. , Checklist of the Quill Mites (Acariformes: Syringophilidae) of the World, Zootaxa. (2015) 3968, no. 1, 1–81, 10.11646/zootaxa.3968.1.1, 26249476. 26249476

[13] Barker F. K. , Cibois A. , Schikler P. , Feinstein J. , and Cracraft J. , Phylogeny and Diversification of the Largest Avian Radiation, Proceedings of the National Academy of Sciences of the United States of America. (2004) 101, no. 30, 11040–11045, 10.1073/pnas.0401892101, 15263073. 15263073 PMC503738

[14] Reddy S. and Cracraft J. , Old World Shrike-Babblers (Pteruthius) Belong With New World Vireos (Vireonidae), Molecular Phylogenetics and Evolution. (2007) 44, no. 3, 1352–1357, 10.1016/j.ympev.2007.02.023, 17412613. 17412613

[15] Irestedt M. and Ohlson J. I. , The Division of the Major Songbird Radiation Into Passerida and ‘Core Corvoidea’ (Aves: Passeriformes) – The Species Tree vs. Gene Trees, Zoologica Scripta. (2008) 37, no. 3, 305–313, 10.1111/j.1463-6409.2007.00321.x.

[16] Cade T. J. , Shrikes as Predators, Proceedings of the Western Foundation of Vertebrate Zoology. (1995) 6, 1–5.

[17] Yosef R. and Pinshow B. , Impaling in True Shrikes (Laniidae): A Behavioral and Ontogenetic Perspective, Behavioural Processes. (2005) 69, no. 3, 363–367, 10.1016/j.beproc.2005.02.023, 15896534. 15896534

[18] Panov E. N. , The True Shrikes (Laniidae) of the World: Ecology, Behavior and Evolution, 2011, Pensoft Publishers.

[19] Yosef R. , Conservation Commentary: Evaluation of the Global Decline in the True Shrikes (Family Laniidae), Auk. (1994) 111, no. 1, 228–233, 10.2307/4088532.

[20] Esely J. D. and Bollinger E. K. , Patterns of Impaling in a Migratory Population of the Loggerhead Shrike, Prairie Naturalist. (2003) 35, 1–8.

[21] Clements J. F. , Schulenberg T. S. , Iliff M. J. , Roberson D. , Fredericks T. A. , Sullivan B. L. , and Wood C. L. , The eBird/Clements Checklist of Birds of the World: v2025, 2025, Cornell Lab of Ornithology, https://www.birds.cornell.edu/clementschecklist/download/.

[22] Winkler D. W. , Billerman S. M. , and Lovette I. J. , Billerman S. M. , Keeney B. K. , Rodewald P. G. , and Schulenberg T. S. , Shrikes (Laniidae), Version 1.0, Birds of the World, 2020, Cornell Lab of Ornithology, 10.2173/bow.laniid1.01.

[23] Skoracki M. , Hromada M. , and Tryjanowski P. , Description of a New Species of Quill Mite Syringophiloidus weiszii sp. n. (Acari, Prostigmata, Syringophilidae) from Great Grey Shrike Lanius Excubitor, Acta Parasitologica. (2001) 46, 30–34.

[24] Skoracki M. , Tryjanowski P. , and Hromada M. , Two New Species of the Genus Syringophilopsis Kethley, 1970 (Acari: Syringophilidae) Parasitising Quills of True Shrikes (Aves: Laniidae), Parasite. (2002) 9, 11–16, 10.1051/parasite/200209111. 11938690

[25] Skoracki M. and Sikora B. , Quill Mites (Acari: Prostigmata: Syringophilidae) From African Passeriform Birds, Zootaxa. (2003) 129, no. 1, 1–10, 10.11646/zootaxa.130.1.1.

[26] Walter D. E. and Krantz G. W. , Krantz G. W. and Walter D. E. , Collecting, Rearing, and Preparing Specimens, A Manual of Acarology, 2009, Texas Tech University Press, 83–96.

[27] Miller E. T. , McTavish E. J. , Gerbracht J. A. , Schloss M. , Iliff M. J. , Lepage D. , Rasmussen P. , and Sullivan B. L. , The Phylogeny of the Birds of the World Tree v1.6/Taxonomy v2025, 2025, Cornell Lab of Ornithology.

[28] Miller E. T. , Sanchez Reyes L. L. , and McTavish E. J. , clootl, 2025, GitHub Repository, https://github.com/eliotmiller/clootl.

[29] McTavish E. J. , Gerbracht J. A. , Holder M. T. , Iliff M. J. , Lepage D. , Rasmussen P. , Redelings B. , Reyes L. L. S. , and Miller E. T. , A Complete and Dynamic Tree of Birds, Proceedings of the National Academy of Sciences of the United States of America. (2025) 122, no. 18, e2409658122, 10.1073/pnas.2409658122, 40299701. 40299701 PMC12067227

[30] Paradis E. and Schliep K. , ape 5.0: An Environment for Modern Phylogenetics and Evolutionary Analyses in R, Bioinformatics. (2019) 35, no. 3, 526–528, 10.1093/bioinformatics/bty633, 30016406. 30016406

[31] Reiczigel J. , Marozzi M. , Fábián I. , and Rózsa L. , Biostatistics for Parasitologists–A Primer to Quantitative Parasitology, Trends in Parasitology. (2019) 35, no. 4, 277–281, 10.1016/j.pt.2019.01.003, 30713051. 30713051

[32] Rózsa L. , Reiczigel J. , and Majoros G. , Quantifying Parasites in Samples of Hosts, Journal of Parasitology. (2000) 86, no. 2, 228–232, 10.1645/0022-3395(2000)086[0228:QPISOH]2.0.CO;2. 10780537

[33] Yosef R. and ISWG International Shrike Working Group , Hoyo J. , Elliott A. , Sargatal J. , Christie D. A. , and Juana E. , Lesser Gray Shrike (Lanius minor), Version 1.0, Birds of the World, 2020, Cornell Lab of Ornithology, 10.2173/bow.legshr2.01.

[34] Yosef R. , ISWG International Shrike Working Group , and Kirwan G. M. , Hoyo J. , Elliott A. , Sargatal J. , Christie D. A. , and Juana E. , Isabelline Shrike (Lanius isabellinus), Version 1.0, Birds of the World, 2020, Cornell Lab of Ornithology, 10.2173/bow.isashr1.01.

[35] Hafner M. S. and Nadler S. A. , Phylogenetic Trees Support the Coevolution of Parasites and Their Hosts, Nature. (1988) 332, no. 6161, 258–259, 10.1038/332258a0. 3347269

[36] Clayton D. H. , Bush S. E. , and Johnson K. P. , Coevolution of Life on Hosts: Integrating Ecology and History, 2015, University of Chicago Press, 10.7208/chicago/9780226302300.001.0001.

[37] Page R. D. M. , Tangled Trees: Phylogeny, Cospeciation, and Coevolution, 2003, University of Chicago Press.

[38] de Vienne D. M. , Refrégier G. , López-Villavicencio M. , Tellier A. , Hood M. E. , and Giraud T. , Cospeciation vs Host-Shift Speciation: Methods for Testing, Evidence from Natural Associations and Relation to Coevolution, New Phytologist. (2013) 198, no. 2, 347–385, 10.1111/nph.12150, 23437795. 23437795

[39] Tryjanowski P. , Hromada M. , and Antczak M. , Breeding Habitat Selection in the Great Grey Shrike Lanius excubitor: The Importance of Meadows and Spring Crops, Acta Ornithologica. (1999) 34, 59–63.

[40] England D. , Breeding the Great Grey Shrike (Lanius Excubitor), Avicultural Magazine. (1971) 77, 1–9.

[41] Clement P. and Worfolk T. , Southern and Eastern Great Grey Shrikes in Northwest Europe, Birding World. (1995) 8, 300–309.

[42] Cramp S. and Perrins C. M. , The Birds of the Western Palearctic Volume 7, 1993, Flycatchers to Shrikes (Oxford University Press.

[43] Yosef R. , ISWG International Shrike Working Group , Sharpe C. J. , Marks J. S. , and Kirwan G. M. , Hoyo J. , Elliott A. , Sargatal J. , Christie D. A. , Juana E. , and Smith M. G. , Great Gray Shrike (Lanius excubitor), Version 1.1, Birds of the World, 2025, Cornell Lab of Ornithology, 10.2173/bow.norshr1.01.1.

[44] Skoracki M. , Sikora B. , and Spicer G. S. , A Review of the Subfamily Picobiinae Johnston and Kethley, 1973 (Acariformes: Prostigmata: Syringophilidae), Zootaxa. (2016) 4113, no. 1, 1–95, 10.11646/zootaxa.4113.1.1, 27395108. 27395108

[45] Jønsson K. A. , Fabre P.-H. , Kennedy J. D. , Holt B. G. , Borregaard M. K. , Rahbek C. , and Fjeldså J. , A Supermatrix Phylogeny of Corvoid Passerine Birds (Aves: Corvides), Molecular Phylogenetics and Evolution. (2016) 94, Pt A, 87–94, 10.1016/j.ympev.2015.08.020, 26327328. 26327328

[46] Aggerbeck M. , Fjeldså J. , Christidis L. , Fabre P.-H. , and Jønsson K. A. , Resolving Deep Lineage Divergences in Core Corvoid Passerine Birds Supports a Proto-Papuan Island Origin, Molecular Phylogenetics and Evolution. (2014) 70, 272–285, 10.1016/j.ympev.2013.09.027, 24125832. 24125832

[47] Skoracki M. , Unsoeld M. , Kosicki J. Z. , Melzer R. R. , Friedrich S. , and Sikora B. , Enigmatic Host-Mite Relationships: Unraveling the Distribution of Quill Mites on Birds-of-Paradise, International Journal for Parasitology. (2024) 54, no. 8-9, 415–427, 10.1016/j.ijpara.2024.03.007, 38575051. 38575051

[48] Skoracki M. , Haarder S. , Unsoeld M. , Nielsen Ó. K. , Hromada M. , and Sikora B. , Syringophilid Mites Parasitising the Crows and the Competitive Exclusion Principle, Scientific Reports. (2025) 15, no. 1, 34436, 10.1038/s41598-025-17527-8, 41038985. 41038985 PMC12491569

